# Preoperative CT Radiomics for Assessing Pathological Response to Neoadjuvant Chemoimmunotherapy in Locally Advanced Non-Small-Cell Lung Cancer

**DOI:** 10.3390/jcm15166150

**Published:** 2026-08-07

**Authors:** Beatrice Trabalza Marinucci, Federica Palmeri, Damiano Caruso, Massimiliano Mancini, Giorgia Piccioni, Fabiana Messa, Anna Maria Ciccone, Giulio Maurizi, Erino Angelo Rendina, Mohsen Ibrahim

**Affiliations:** 1 Department of Thoracic Surgery, Sant’Andrea Hospital, Sapienza University, 00189 Rome, Italy; giorgia.piccioni@uniroma1.it (G.P.); fabiana.messa@uniroma1.it (F.M.); aciccone@ospedalesantandrea.it (A.M.C.); giulio.maurizi@uniroma1.it (G.M.); erinoangelo.rendina@uniroma1.it (E.A.R.); 2 Department of Radiology, Sant’Andrea Hospital, Sapienza University, 00189 Rome, Italy; federica.palmeri@uniroma1.it (F.P.); damiano.caruso@uniroma1.it (D.C.); 3 Department of Morphologic and Molecular Pathology, Sant’Andrea Hospital, Sapienza University, 00189 Rome, Italy; mamancini@ospedalesantandrea.it

**Keywords:** non-small-cell lung cancer, neoadjuvant chemoimmunotherapy, locally advanced disease, radiomics

## Abstract

**Background/Objectives**: Reliable preoperative assessment of treatment response after neoadjuvant chemoimmunotherapy in locally advanced non-small-cell lung cancer (NSCLC) remains challenging because conventional imaging may not accurately distinguish viable tumour from treatment-related fibrosis or immune-mediated changes. This study investigated whether CT-derived radiomic features combined with machine learning could improve the identification of patients achieving pathological complete response (pCR). **Methods**: Twenty-nine consecutive patients with stage III NSCLC who underwent surgical resection following neoadjuvant chemoimmunotherapy were retrospectively analysed. Radiomic features were extracted from preoperative CT scans and used to develop supervised machine-learning models based on Random Forest, Support Vector Machine, K-Nearest Neighbors, Multi-Layer Perceptron, and Logistic Regression algorithms. Histopathological findings after surgery served as the reference standard. Feature distributions were compared between patients with and without pCR using the Mann–Whitney U test with Bonferroni correction. **Results**: Surgical procedures included 19 lobectomies, 2 bilobectomies, 3 pneumonectomies, and 5 complex major resections. Pathological complete response was observed in 9 of 29 patients (31%). The proposed radiomics-based model achieved an area under the ROC curve of 0.92 (95% CI, 0.81–1.00), with 91% accuracy, 90% sensitivity, and 92% specificity (*p* < 0.05). All patients classified by the model as complete responders were confirmed to have pathological complete response at postoperative histological examination. **Conclusions**: CT-based radiomics combined with machine learning demonstrated promising performance for the preoperative prediction of pathological response after neoadjuvant chemoimmunotherapy in stage III NSCLC. Although these findings require external validation in larger prospective cohorts, this approach may support preoperative treatment assessment and surgical decision-making.

## 1. Introduction

Non–small-cell lung cancer (NSCLC) accounts for around 80–85% of all lung cancer diagnoses. Nearly 20% of patients present with stage IIIA–IIIB disease at diagnosis [1]. Surgery remains the cornerstone of treatment with the intention of cure for both early-stage and selected cases of locally advanced NSCLC. Nevertheless, the optimal management of stage III disease remains controversial. Stage III NSCLC encompasses a highly heterogeneous group of diseases characterised by different combinations of primary tumour extension and mediastinal lymph node involvement, which makes treatment decisions particularly complex and requires a multidisciplinary approach. Although stage IIIB disease is traditionally considered unresectable, some patients may still be eligible for surgery after careful evaluation by a multidisciplinary tumour board, thus highlighting the importance of personalised treatment strategies [2].

Over the last few years, substantial changes have occurred in the therapeutic landscape of locally advanced NSCLC. Several landmark clinical trials have demonstrated that adding immune checkpoint inhibitors (ICIs) to platinum-based chemotherapy in the neoadjuvant setting significantly improves pathological response rates and survival outcomes in intention-to-treat populations [3]. As neoadjuvant chemoimmunotherapy becomes more widely adopted in clinical practice, traditional concepts of resectability are being re-evaluated. Advances in systemic therapies have increased the number of patients who are potentially eligible for surgery and raised new questions regarding patients’ selection, the timing of surgery, and the operative decision-making.

Despite these advances, accurately assessing resectability after neoadjuvant chemoimmunotherapy remains challenging. Clinical decision-making is often complicated by the risk of understaging or overstaging of residual disease, the possibility of disease progression during systemic treatment, and treatment-related toxicities. Nevertheless, evidence from multiple clinical trials has shown that the adverse events associated with neoadjuvant chemoimmunotherapy treatment are generally manageable. This allows most medically operable patients with resectable N2 disease to proceed to surgery without significant delay. Consequently, patients with more advanced-stage tumours, particularly those with high programmed death ligand 1 (PD-L1) expression, are increasingly being considered for neoadjuvant chemoimmunotherapy, rather than upfront surgery [2,4].

A critical unresolved issue is how to assess residual disease following neoadjuvant treatment. Although radiological downstaging is often observed after chemo-immunotherapy, the extent to which this reflects pathological response remains unclear. This uncertainty is particularly relevant in patients with borderline resectable tumours, where treatment-induced changes may influence surgical planning. Indeed, fibrosis, inflammation and immune cell infiltration induced by the treatment itself can make surgical dissection technically challenging and may mimic persistent disease on imaging studies. Currently, there is no reliable diagnostic tool that can consistently distinguish between viable tumour tissue and treatment-related fibrotic changes before surgery. This limits our ability to accurately predict pathological response and the true resectability after NA chemo-immunotherapy.

In this evolving scenario, radiomics has emerged as a promising approach to overcoming some of the limitations of conventional imaging assessments. It is a methodology that uses advanced quantitative imaging techniques and artificial intelligence (AI) algorithms to extract a variety of quantitative features from routinely acquired medical images, such as computed tomography (CT), magnetic resonance imaging (MRI) and positron emission tomography (PET) scans. These features capture information relating to tumour shape, texture, intensity, spatial heterogeneity and other imaging characteristics that cannot be discerned through visual assessment alone [5].

In recent years, the application of radiomics in thoracic oncology has expanded considerably, demonstrating its potential value in diagnosis, prognosis, assessing treatment responses, and predicting clinical outcomes [6,7]. This growing interest partly stems from the recognised limitations of conventional radiological response criteria. Although Response Evaluation Criteria in Solid Tumours (RECIST) remain the standard method for evaluating treatment response, they rely primarily on changes in tumour size, and do not adequately capture the complex biological effects induced by immunotherapy. Furthermore, the RECIST criteria may fail to account for non-measurable lesions, immune-related inflammatory changes or pseudo-progression, whereby lesions initially increase in size before subsequently regressing [8]. Consequently, residual radiological abnormalities following neoadjuvant treatment may not accurately reflect viable tumour burden.

Radiomics offers the possibility of extracting biologically meaningful information from imaging data that could more accurately reflect the tumour’s underlying pathological status. Leveraging machine-learning algorithms applied to CT images can improve the prediction of pathological complete response (pCR) following neoadjuvant chemoimmunotherapy, providing clinicians with a non-invasive tool to support treatment planning and surgical decision-making [5,9].

The present study therefore aimed to investigate whether CT-derived radiomic features combined with supervised machine-learning algorithms could improve the preoperative identification of patients who will achieve a pCR following neoadjuvant chemoimmunotherapy for stage III NSCLC. A reliable, non-invasive imaging biomarker that can predict pCR before surgery could improve the assessment of treatment response, support surgical planning and facilitate multidisciplinary decision-making.

## 2. Materials and Methods

### 2.1. Patients

Pre-operative CT scans of 29 patients who underwent surgery between February 2023 and May 2025 following NA ICI-CHT at a single institution were included in the study. Each image contained exactly one volume of interest (VOI), ensuring univocal correspondence between subjects and samples.

The inclusion criteria for the dataset were as follows: patients affected by NSCLC stage IIIA-IIIB (TNM 8th classification); patients undergoing four cycles of neoadjuvant chemoimmunotherapy; patients undergoing total-body CT scanning with medium contrast and endobronchial ultrasound (EBUS) for restaging after therapy; and patients eligible for surgery after restaging.

Exclusion criteria: patients with metastatic disease from the outset; patients with oncogene-addicted tumours (including EGFR-mutated tumours); patients with a low performance status or severe comorbidities who were not eligible for surgery; patients who had to discontinue neoadjuvant therapy prematurely due to toxicity; and patients who had experienced metastatic progression. EGFR-mutated tumours were excluded from treatment with neoadjuvant chemoimmunotherapy according to the selection criteria applied to our cohort. Therefore, EGFR mutation status was not included as a potential confounder in the present analysis. Due to the limited sample size, PD-L1 expression was not incorporated as an independent predictor in the machine-learning model to avoid overfitting.

The dataset of included patients was used to train, validate, and test different machine-learning models. A robust radiomic approach was applied to detect two groups: complete responders and non-complete responders, according to final pathology. The cohort consisted of 9 complete response cases and 20 non-complete response cases.

### 2.2. CT Scan Protocol

Chest computed tomography (CT) scans were performed with patients lying on their back and holding their breath at full inspiration, to minimise respiratory motion artefacts and ensure optimal lung expansion. Image acquisition was carried out in the cranio–caudal direction following the intravenous administration of an iodinated contrast medium, in accordance with institutional protocols.

All scans were acquired using a 128-detector-row CT scanner (Revolution EVO, GE Medical Systems, Chicago, IL, USA). The standardised acquisition protocol included a tube voltage of 120 kVp and automatic tube current modulation ranging from 100 to 250 mAs, which allowed for the optimisation of image quality whilst reducing radiation exposure. Helical acquisition was performed with a pitch of 0.98 and a detector collimation of 0.625 mm to enable high-resolution volumetric imaging of the thorax.

Raw projection data were reconstructed using an adaptive statistical iterative reconstruction algorithm (ASiR-V, blending level 50%; GE Medical Systems), which improves image quality by reducing image noise while preserving spatial resolution. Reconstructed images were generated with a slice thickness of 0.5 mm, providing thin-section datasets suitable for detailed anatomical assessment and subsequent radiomic feature extraction [10].

### 2.3. Radiomic-Based Machine-Learning Modelling

Radiomic analysis was performed in accordance with the Image Biomarker Standardization Initiative (IBSI) recommendations, ensuring methodological standardisation and reproducibility throughout the image processing and feature extraction workflow [11].

Deep learning-derived features were extracted using the automated Trace4Research^TM^ platform (version 2.1.00, DeepTrace Technologies S.r.l., Milan, Italy), which provides a standardized IBSI-compliant radiomics pipeline. The model was used exclusively for feature extraction and was neither trained nor fine-tuned on the present cohort. Image preprocessing, normalization, resizing, and feature generation were performed automatically within the platform according to its validated workflow, ensuring consistent processing across all included patients. The workflow included VOI segmentation, image preprocessing, radiomic feature extraction, feature selection, machine-learning model development, validation, testing, and calibration analysis.

The primary tumour volume of interest (VOI) segmentation was manually performed on contrast-enhanced CT images by an experienced thoracic radiologist via slice-by-slice contouring of the lesion. Following segmentation, image-preprocessing procedures were applied to reduce variability related to image acquisition and reconstruction. Image preprocessing included isotropic resampling by linear interpolation. Specifically, image intensities were normalised and all images were resampled to isotropic voxel spacing to ensure feature comparability across the dataset.

Radiomic feature extraction was subsequently performed on the segmented VOIs. IBSI-compliant handcrafted radiomic features were extracted from the original and filtered images, including Morphology describing tumour shape and geometry, Intensity-based Statistics, Intensity Histogram, Gray-Level Co-occurrence Matrix (GLCM), Gray-Level Run Length Matrix (GLRLM), Gray-Level Size Zone Matrix (GLSZM), Neighbourhood Gray-Tone Difference Matrix (NGTDM), and Gray-Level Dependence Matrix (GLDM) features, with intensity discretization performed using a fixed bin width of 25.

The platform also enabled extraction of deep-learning-derived features through the convolutional layers of a pretrained ResNet50 model. Specifically, 2048 deep features were generated from images discretized into 256 bins and resampled to 224 × 224 × 28 voxels. These Deep Features, as defined in the manuscript, represent an additional feature class derived from a pretrained convolutional neural network and are not included in the IBSI framework.

Handcrafted radiomic features quantify predefined tumour characteristics, including intensity, shape, and texture, and may serve as indirect-imaging surrogates of intra-tumoral heterogeneity related to cellularity, necrosis, fibrosis, vascularization, and immune infiltration. These associations remain biologically plausible hypotheses, rather than direct histopathological correlates. Compared with deep learning-derived features, handcrafted features are more interpretable, whereas deep learning may capture complementary, non-linear imaging patterns. Given the high dimensionality of the radiomic dataset, a feature-selection procedure was performed to reduce redundancy, minimize the risk of overfitting, and retain the most informative variables for classification. The selection process included the removal of highly correlated features, statistical assessment of feature relevance with respect to treatment response, and optimization procedures aimed at maximizing the predictive information content of the final feature set. Following dimensionality reduction, 149 features were retained and used for model development.

The 2860 features constituted the initial high-dimensional feature space, whereas the 149 features represented a candidate pool obtained after removing redundant or non-informative variables. Feature reduction was performed within the platform by removing low-variability and low-information features, followed by a mutual-information-based optimization procedure to minimize redundancy. Model performance was subsequently assessed using nested cross-validation to limit information leakage and reduce the risk of overfitting.

A machine-learning analysis was conducted using a supervised learning approach, with the pathological response to treatment serving as the reference standard. The objective was to perform a binary classification of patients who achieved a pathological complete response (pCR) and those with residual viable tumour. Five supervised machine-learning architectures were evaluated using nested random 7-fold cross-validation: Random Forest (RF), Support Vector Machine (SVM), K-Nearest Neighbors (KNN), Multi-Layer Perceptron (MLP) and Logistic Regression (LR). Hyper-parameter optimization was performed by grid search, and all preprocessing steps, including Adaptive Synthetic Sampling (ADASYN) oversampling, normalization, and Principal Component Analysis (PCA) optimization, were performed after data splitting to avoid information leakage.

The selected radiomic features were then used to train, validate and test the classifiers, thus enabling the development of predictive models to identify complete responders following neoadjuvant chemoimmunotherapy.

### 2.4. Model Development and Validation

Five machine-learning classifiers were trained: Random Forest, Support Vector Machine, K-Nearest Neighbors, Multi-Layer Perceptron, and Logistic Regression.

Each model was implemented using ensemble strategies and evaluated through nested cross-validation. Dimensionality reduction techniques such as Principal Component Analysis (PCA) and Fisher Discriminant Ratio (FDR) were also explored.

Model performance was evaluated using standard classification metrics, including receiver operating characteristic area under the curve (ROC-AUC), accuracy, sensitivity, specificity, positive predictive value (PPV), negative predictive value (NPV), and F1-score, with confidence intervals and statistical testing. Model calibration was assessed using reliability diagrams, Expected Calibration Error, and Brier Score (BS).

No independent holdout test cohort was created, and the 29 patients were not permanently divided into separate training, validation, and testing subsets. The reported training, validation, and internal testing results were generated within the nested cross-validation framework.

Specifically, the outer cross-validation loop provided the internal test folds used to estimate model performance, while the inner loop was used for feature selection, dimensionality reduction, and model optimization using only the training portion of each outer iteration. Therefore, each patient contributed to internal testing only when included in an outer test fold, and was not used for model fitting or optimization during that iteration. The final internal test performance represents the aggregation of these out-of-fold predictions across the outer folds.

### 2.5. Surgery and Pathology

Pathology was primarily ascertained through biopsies of the lung or bronchi. Central tumours required bronchoscopy to assess potential endobronchial invasion. If N1/N2 staging was suspected clinically, patients underwent EBUS with eventual trans-bronchial needle aspiration (TBNA) or mediastinoscopy.

Pathological assessments included routine immunohistochemistry and genetic testing for EGFR mutations.

Patients received two different protocols of neoadjuvant therapy according to their pathology: those affected by adenocarcinoma received carboplatin, pemetrexed and pembrolizumab, while those affected by squamous cell carcinoma received carboplatin, paclitaxel (or nab-paclitaxel) and pembrolizumab.

After 4 cycles of neoadjuvant therapy, all patients underwent clinical re-staging by Total Body-CT with contrast medium and PET, and EBUS. Patients who had undergone clinical downstaging after NA therapy and were fit for surgery underwent lung resection following a multidisciplinary discussion of their case. The stage of the tumours was classified according to the 8th TNM Staging System.

All patients were operated on at the same centre. In all cases, surgery was performed using the same approach: a muscle-sparing lateral thoracotomy involving a 5–6 cm incision in the fifth intercostal space. The surgical strategy aimed for R0 resection with lung preservation wherever possible. Procedures ranged from segmentectomy and standard lobectomy to bilobectomy, pneumonectomy, and major reconstructions involving the bronchus, carina, pulmonary artery or thoracic aorta. Reconstructive techniques for locally advanced lung cancer, such as sleeve resection of the bronchus, pulmonary artery or carina, and thoracic aorta reconstruction, were applied when necessary.

Systematic mediastinal lymph node dissection was performed in all cases.

The postoperative pathology was confirmed by examining surgical resection samples collected after neoadjuvant immune-chemotherapy. All surgical specimens were processed according to institutional thoracic pathology protocols and international guidelines [12], with extensive tumour-bed sampling to estimate residual viable tumour.

Pathologic complete response (PCR) was defined as the absence of any viable tumour in both the primary site and sampled lymph nodes; major pathologic response (MPR) was defined as 10% or less viable residual tumour cells.

### 2.6. Statistical Analysis

Data were prospectively collected and stored in an Excel database (Version 16.0, Microsoft Corp, Redmond, WA, USA). Categorical data were described with the absolute and relative (%) frequency, continuous data were summarized with the mean and standard deviation (SD). Baseline characteristics were compared between the 2 groups (Complete Responders and non-Complete Responders): quantitative variables were compared using the Student’s *t*-test, whereas categorical variables were compared using the Fisher–Freeman–Halton exact test.

All statistical analyses were performed using the analytical tools embedded within the Trace4Research^TM^ platform. Descriptive statistics were used to characterise the distribution of the most relevant radiomic features in the two study groups: patients who achieved a pathological complete response (pCR) and patients with residual disease after neoadjuvant treatment. As radiomic features generally do not follow a normal distribution, continuous variables were summarised using median values and corresponding 95% confidence intervals (95% CI).

To investigate the discriminatory ability of individual radiomic features, a univariate analysis was performed by comparing the distributions of features between complete and non-complete responders. As radiomic data is not normally distributed, comparisons were conducted using the non-parametric Wilcoxon rank-sum test (Mann–Whitney U test), which evaluates whether the distributions of a given feature differ significantly between two independent groups.

As a large number of radiomic variables were analysed simultaneously, correction for multiple testing was applied to reduce the risk of type I errors (false positives). Specifically, *p*-values obtained from the univariate analyses were adjusted using the Bonferroni correction method. Statistical significance was defined as an adjusted two-sided *p*-value of less than 0.05.

Although no individual radiomic feature remained statistically significant after Bonferroni correction, all extracted features were considered as candidate predictors and were evaluated through the feature selection process for machine-learning model development.

### 2.7. Consent and Ethics

Written informed consent from all participants was obtained. The study was conducted in accordance with the Declaration of Helsinki and local institutional governance procedures (Prot. n. 7 SA_2023 RIF. CE 7031/2022). The study protocol was developed beginning in February 2023, whereas patient enrolment started only after institutional ethics committee approval, obtained on 1 February 2023. Consecutive eligible patients were prospectively enrolled from that date onward in full compliance with ethical requirements, while the analysis of clinical and imaging data was performed retrospectively after completion of the enrolment period.

Clinical and aggregate data supporting the study findings are available in the manuscript. Patient-level data and imaging-derived datasets are not publicly available because of privacy, ethical, and institutional restrictions. The radiomic feature matrix, preprocessing settings, model parameters, and final specifications were generated within the Trace4Research™ platform and may be made available in anonymized form upon reasonable request, subject to institutional approval and applicable data-protection regulations.

## 3. Results

### 3.1. Clinical Characteristics

Clinical stage at presentation was IIIA in 14 (48%) and IIIB in 15 (52%). Adenocarcinoma represented the predominant histologic subtype (22, 75.9%), whereas squamous cell carcinoma accounted for the remaining cases (7, 24.1%). The cohort included 16 (55.2%) men and 13 (44.8%) women, with an overall mean age of 65.1 ± 11.5 years. All patients had clinical downstaging and were considered fit for surgery.

Operative procedures included the following: 19 (66%) standard lobectomies, 2 (7%) bilobectomies, 3 (10%) pneumonectomies, 5 (17%) reconstructive surgery (2 sleeve of the pulmonary artery, 1 carinal right upper-sleeve lobectomy, and 2 lobectomies with aortic reconstruction).

Pathological outcomes were notable. PCR at final pathology was documented in nine (31%) patients. Nine (31%) patients showed downstaging to stage II, and 11 (38%) patients showed downstaging to stage I.

Nodal clearance to ypN0 was observed in 22 patients (76%). Particularly, pre-treatment N2 involvement was described in 18 (62%), while at final pathology pathological nodal staging (ypN) showed ypN0 in 22 patients (76%), ypN1 in 3 (10%), and ypN2 in 4 (14%).

The final post-treatment pathological stage distribution included nine stage 0 cases (complete response, 31%), four stage IIIA (14%), five stage IIB (17%), three stage IB (10%), and eight stage IA (28%).

Pre-operative baseline characteristics of the population are described in Table 1. These have been compared between complete responders and non-complete responders’ patients.

### 3.2. Radiomics Features and ROC Curves

A total of 2860 features were extracted from each segmented volume of interest (VOI) in each image. Of these, 149 were selected for model training. These features were then used to validate and test the five different machine-learning classifiers considered in this study.

**Figure 1:**
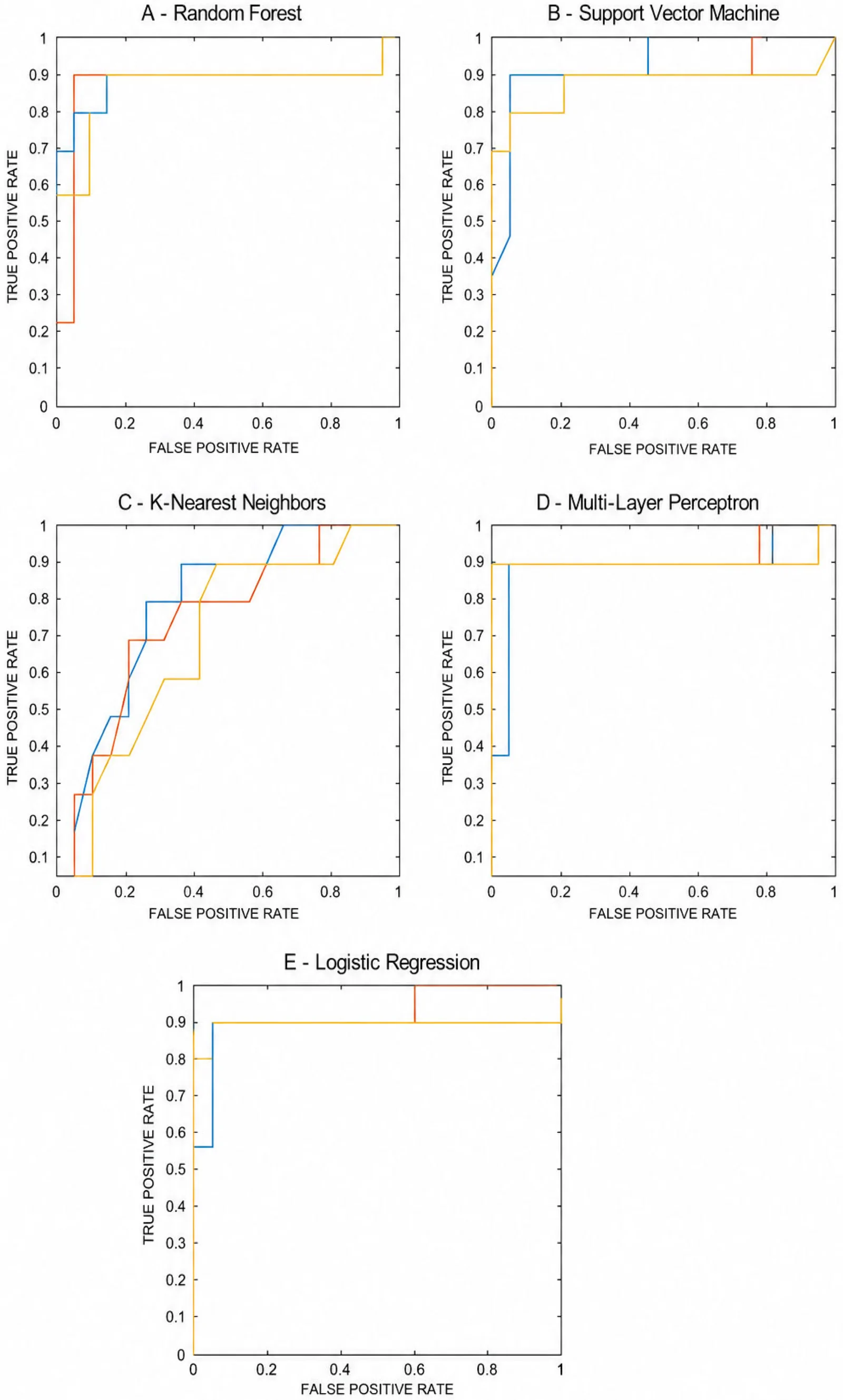
Performance of the five machine-learning classifiers within the nested cross-validation framework. Each box corresponds to a different classifier: Random Forest (RF), Support Vector Machine (SVM), K-Nearest Neighbors (KNN), Multi-Layer Perceptron (MLP), and Logistic Regression (LR). Within each box, the different line types represent the results obtained during training, validation, and internal testing.

ROC curve analysis revealed a high level of discrimination across all models. The ROC-AUC values for the five models are plotted in Figure 1A–E. Of the tested algorithms, the Multi-Layer Perceptron (MLP) classifier performed the best overall and was the most effective model for the binary classification task. Specifically, the MLP yielded a ROC-AUC of 0.92 (95% CI: 0.81–1.00), indicating excellent discriminatory capability. Additionally, the model achieved an overall accuracy of 91% (95% CI: 84–98%), correctly classifying the majority of patients. The sensitivity was 90% (95% CI: 79–100%), reflecting the model’s ability to correctly identify patients who ultimately achieved pathological complete response (pCR), while the specificity was 92% (95% CI: 84–100%), indicating a high capacity to correctly classify patients with residual viable tumour. Overall model performance was statistically significant (*p* < 0.05) (see Table 2). Table 3 outlines the model’s performance detailed by patient sex stratification.

**Figure 2:**
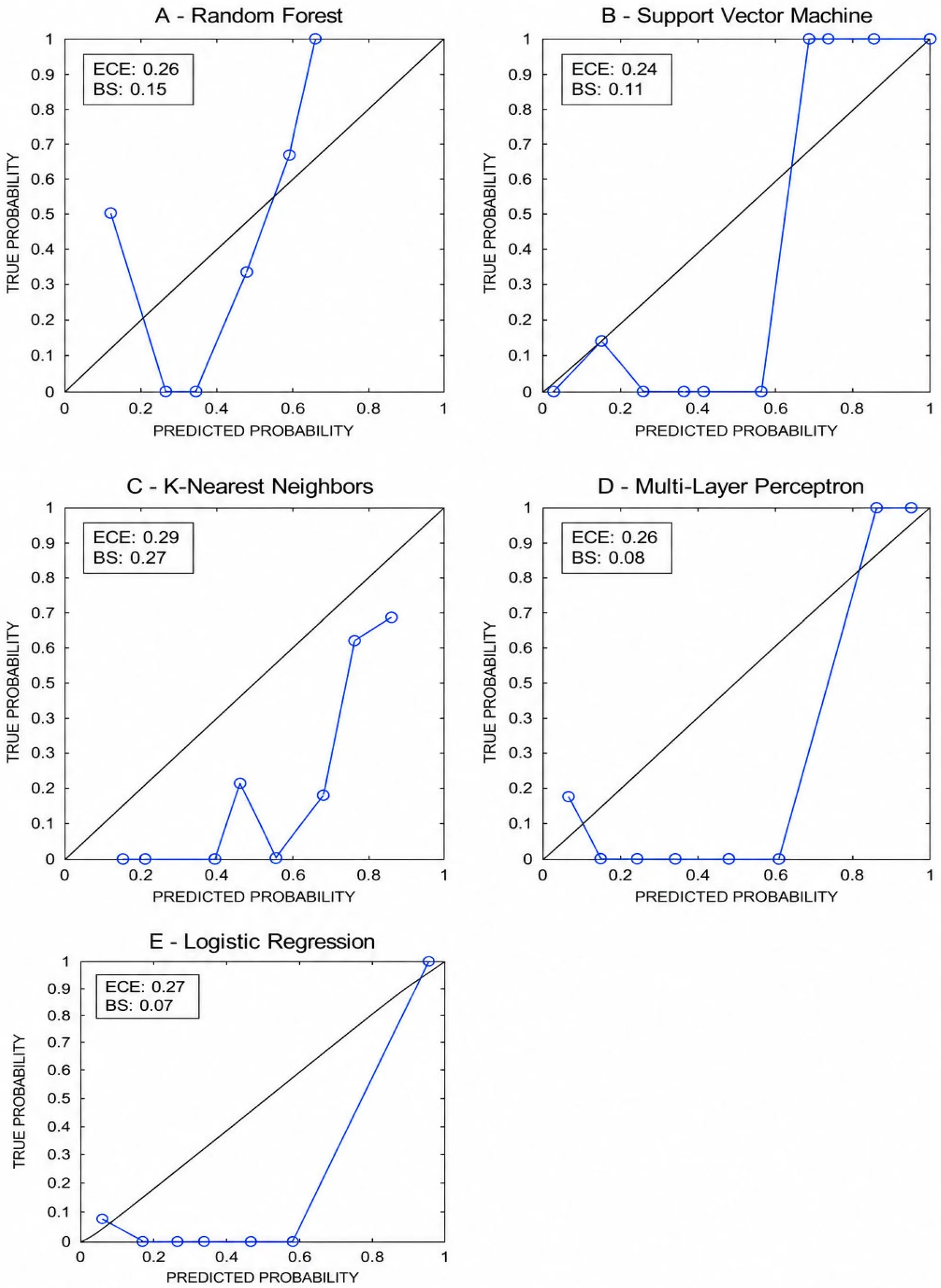
Probability diagrams for each model from internal testing. Predicted probability is compared with true probability of being in the positive class. ECE and BS are reported.

Calibration plots for all machine-learning classifiers are shown in Figure 2. Model calibration was assessed by comparing predicted and observed probabilities of pathological complete response and quantified using the Expected Calibration Error (ECE) and Brier Score (BS).

Following feature selection, 149 radiomic and deep learning-derived features were retained for model development. Analysis of the feature importance of the best-performing classifier identified the 40 most relevant predictors, which were then ranked according to their contribution to model performance. The median values, 95% confidence intervals, and adjusted *p*-values from Wilcoxon rank-sum tests are reported for each selected feature (Table 4). Texture-related features derived from GLCM, GLRLM, GLSZM, and NGTDM matrices, as well as selected deep learning-based descriptors, were among the most informative predictors.

## 4. Discussion

Surgery remains the cornerstone of curative treatment for early-stage and locally advanced NSCLC, with five-year overall survival rates ranging from 92% in stage IA to 26% in stage IIIB [13]. Neoadjuvant chemoimmunotherapy has recently gained considerable attention in the management of resectable NSCLC, as it can reduce tumour burden and potentially improve survival outcomes. The strong and consistent association between pathological response and survival following neoadjuvant therapy supports the use of pathological complete response (pCR) and major pathological response (MPR) as surrogate endpoints for overall survival in this setting [14].

The management of stage III NSCLC remains controversial among surgeons and centres, particularly in cases involving multi-station N2 disease, bulky N2 involvement and T4 invasion. Recent advances in NA ICI-CHT have introduced additional complexity, as treatment response can significantly impact resectability. Notably, some patients achieve significant responses to neoadjuvant chemoimmunotherapy, which can make previously unresectable tumours amenable to surgery. In fact, several studies report an R0 resection rate of 70–85% in patients considered borderline resectable, suggesting that the decision to operate should be based on the patient’s response to therapy, rather than prior to therapy. In the recently published Expert Consensus Document, the expert panel considered surgical resection a reasonable option for selected patients with T4N2 disease characterised by tumours larger than 7 cm, provided an R0 resection is deemed achievable based on tumour location. Similarly, patients with T4N2 disease resulting from invasion of structures that can be resected with limited additional morbidity should undergo multidisciplinary evaluation to assess surgical candidacy [15].

The presence of N2 disease, including persistent nodal involvement after initial treatment, does not necessarily rule out lung-sparing surgery or automatically necessitate pneumonectomy. Data from clinical trials evaluating neoadjuvant chemoimmunotherapy consistently demonstrate favourable surgical outcomes, characterised by high R0 resection rates and low pneumonectomy rates, typically below 12%. Importantly, when pneumonectomy is required to achieve complete resection, patients appear to benefit from neoadjuvant chemoimmunotherapy in a similar way to those undergoing lobectomy [16,17].

The question of real resectability following a radiographic response remains unanswered. For patients with borderline resectability due to anatomical tumour location, it is unclear whether radiographic downstaging translates into improved resectability. Furthermore, fibrosis induced by neoadjuvant chemoimmunotherapy can obscure dissection planes, making surgery technically challenging and hindering the reliable differentiation between fibrosis and residual viable tumour during surgery [18].

In addition, there is currently no consensus on how to best assess pathological response after neoadjuvant chemoimmunotherapy. Patients treated with an intent-to-treat approach are usually evaluated based on their clinical response, as determined by imaging. However, caution must be exercised when interpreting imaging findings, as pseudo progression and reactive uptake on PET-CT can occur due to treatment-induced inflammatory changes and fibrosis [19]. Although imaging criteria such as RECIST 1.1 remain widely used for response evaluation, discrepancies between radiographic and histopathological findings are well documented, particularly in the context of neoadjuvant immunotherapy. Residual radiographic abnormalities may not accurately reflect viable tumour burden, as they can represent fibrosis or immune-related changes. This discordance highlights the need for histopathological confirmation to inform clinical decision-making and prevent premature conclusions. Consequently, the identification of reliable predictive biomarkers of response has become a major focus of research. In this context, radiomics has emerged as a promising non-invasive approach for predicting the efficacy of neoadjuvant chemoimmunotherapy in resectable NSCLC. Radiomics enables the extraction of high-dimensional quantitative features from imaging data, allowing the development of predictive models that extend beyond conventional visual assessment. For example, Wang et al. demonstrated that pretreatment CT-based radiomic features could predict MPR following neoadjuvant chemoimmunotherapy, achieving an AUC of 0.60 [20,21].

To the best of our knowledge, the present study represents the first Italian experience investigating the application of radiomics in a clinically relevant setting such as preoperative stratification of response to neoadjuvant therapy in NSCLC. The use of a standardized analytical pipeline and the integration of multiple machine-learning algorithms enhance the robustness of the proposed model.

**Figure 3:**
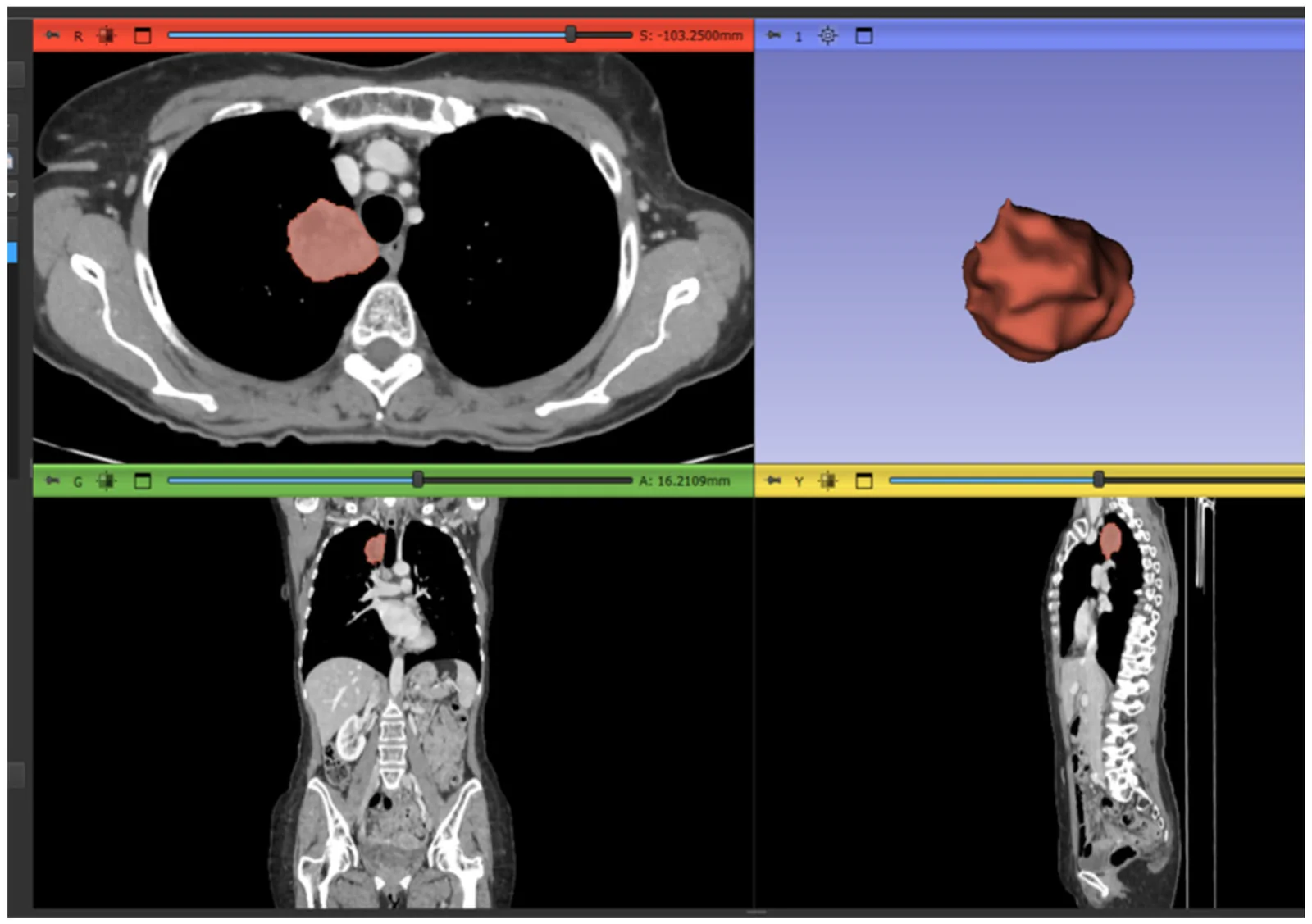
Radiomic-based machine-learning modelling.

Our findings suggest that a radiomics-based machine-learning approach can accurately predict how patients will respond to treatment using preoperative CT imaging (Figure 3). The consistently high performance across different classifiers suggests that radiomic features capture clinically meaningful information related to tumour biology. Notably, the models demonstrated a strong ability to discriminate between complete and non-complete responders.

Of the machine-learning algorithms evaluated, the Multi-Layer Perceptron (MLP) demonstrated the best predictive performance, achieving an area under the receiver operating characteristic curve (ROC-AUC) of 0.92 (95% confidence interval CI: 0.81–1.00). The model also exhibited high overall accuracy (91%, 95% CI: 84–98%), with a sensitivity of 90% (95% CI: 79–100%) and a specificity of 92% (95% CI: 84–100%) (*p* < 0.05). These results suggest that the model has an excellent ability to distinguish between patients who achieved a pathological complete response (pCR) following neoadjuvant chemoimmunotherapy and those with residual viable tumour at surgical pathology.

Notably, all nine patients classified as complete responders by the radiomic model were confirmed to have achieved a pathological complete response (pCR) on final histopathological examination. While the limited sample size warrants cautious interpretation, this result suggests a very high positive predictive value, and highlights the potential of radiomics as a non-invasive biomarker of treatment response. Accurate identification of pCR before surgery is crucial in the management of locally advanced NSCLC treated with neoadjuvant chemoimmunotherapy, as it is currently one of the major unmet needs. Current imaging modalities often struggle to distinguish viable tumour tissue from treatment-induced fibrosis, necrosis, inflammation or immune-cell infiltration, all of which may persist despite the complete eradication of malignant cells [22].

The high level of agreement observed between radiomics-based predictions and pathological findings supports the hypothesis that quantitative imaging biomarkers may capture biological information that cannot be detected through conventional radiological assessment alone. Furthermore, radiomic analysis examines tumour morphology, intensity distributions and intra-tumoral heterogeneity in much greater detail, providing a more accurate representation of the underlying biological response to treatment [20]. Integrating handcrafted radiomic features with deep learning-derived descriptors appears to provide complementary information, thereby enhancing predictive performance.

From a clinical perspective, these findings are particularly relevant because they could help to refine surgical decision-making after neoadjuvant therapy. Reliable prediction of pCR could improve patient selection for surgery, facilitate multidisciplinary discussions regarding resectability, and support decisions concerning the optimal extent of resection. In borderline cases where residual radiological abnormalities could represent either persistent disease or treatment-related fibrosis, radiomics could provide information to inform operative planning. Moreover, a more precise evaluation of the response to treatment could potentially impact the timing of surgery and mitigate uncertainty in post-neoadjuvant management [21].

The strong performance of the proposed model likely reflects the complementary contributions of handcrafted radiomic features and deep learning-derived imaging descriptors. Handcrafted features quantify predefined image characteristics, such as tumour shape, intensity and texture, whereas deep learning features can automatically identify complex, potentially non-intuitive imaging patterns. Integrating these two approaches may therefore provide a more comprehensive characterisation of tumour phenotype. It is notable that texture-related features emerged as some of the most informative predictors, suggesting that intra-tumoral heterogeneity may be closely associated with sensitivity to neoadjuvant chemoimmunotherapy and the likelihood of achieving a complete pathological response.

Nevertheless, considering the limited cohort of 29 patients and 9 pathological complete responses, the findings should be regarded as exploratory and hypothesis-generating. Internal cross-validation can reduce, but cannot eliminate, the risk of model instability and optimistic performance estimates in a small dataset. Therefore, validation in larger independent cohorts remains necessary to establish the robustness and generalizability of the proposed radiomic approach.

This study has several limitations. First, the relatively small sample size increases the risk of overfitting and limits the generalizability of the findings. Although feature selection and internal validation procedures were implemented to mitigate this risk, the reproducibility and robustness of the model need to be confirmed by larger, multicentre cohorts. Given the limited sample size and the imbalance between complete and non-complete responders, the reported predictive performance should be interpreted with caution. Therefore, the proposed radiomics-based approach should be considered exploratory, and requires confirmation in larger external cohorts.

Second, the study was conducted within a single-centre setting, potentially limiting the generalizability of the findings to other institutions with different patient populations, imaging equipment, or acquisition protocols. Variability in image acquisition and reconstruction parameters is a well-recognized challenge in radiomics, and may affect feature stability and model performance.

Future investigations should therefore focus on external validation in large multicentre cohorts and on the harmonization of imaging acquisition, reconstruction, and radiomic analysis workflows.

An additional limitation is the potential selection bias arising from the inclusion of only patients who successfully completed neoadjuvant chemoimmunotherapy and subsequently underwent surgical resection. Because pathological complete response can only be assessed in resected specimens, the present model was intentionally developed in this selected population. Consequently, its applicability to the broader population of patients receiving neoadjuvant chemoimmunotherapy, including those who experience disease progression, treatment-related toxicity, or remain unsuitable for surgery, remains uncertain.

The present exploratory study was designed to assess the feasibility of CT-based radiomics for predicting pathological complete response after neoadjuvant chemoimmunotherapy, rather than to compare its performance with conventional response-assessment methods. Given the limited sample size, developing reliable comparative or integrated models including clinical, pathological, metabolic, and radiological variables was not feasible. We acknowledge that evaluating the incremental value of radiomics beyond established predictors is clinically relevant and should be addressed in future larger, preferably multicentre, studies comparing conventional, radiomic, and combined models.

Furthermore, the biological interpretation of the identified radiomic and deep-learning features remains incomplete. In the present study, the primary objective was predictive modelling of pathological complete response, rather than radiogenomic or radiopathological correlation. Therefore, a systematic correlation between individual radiomic features and specific histopathological components was not performed. Future radiopathological correlation studies integrating quantitative-imaging biomarkers with detailed histopathological assessment of fibrosis, necrosis, immune-cell infiltration, and residual viable tumour may help elucidate the biological mechanisms captured by radiomic signatures.

The Bonferroni-adjusted analyses reported in Table 4 were performed as exploratory univariate comparisons to assess the individual discriminatory ability of each feature. None of the 40 listed features reached statistical significance after Bonferroni correction; therefore, no single radiomic feature can be considered independently associated with pathological complete response on the basis of these analyses. Bonferroni-adjusted *p*-values of 1.00 reflect the high-dimensional nature of the data, correlated radiomic features, and limited statistical power; therefore, no individual feature remained significant after correction, and the univariate results should be considered exploratory. We also acknowledge that the female subgroup was too small, making the observed AUC of 100% likely a sample-specific finding, rather than evidence of perfect performance. Finally, the wide confidence intervals for sensitivity and specificity highlight the uncertainty related to the limited sample size, emphasizing that the reported performance requires validation in larger independent cohorts.

The machine-learning feature-selection process was not based exclusively on univariate statistical significance. The 149 features represented a candidate multivariable feature pool identified through the removal of redundant and highly correlated variables and through feature-ranking and dimensionality-reduction procedures. In radiomics, features with limited discriminatory ability when evaluated individually may still provide complementary information when considered jointly within a multivariable model, particularly when they characterize different aspects of tumour morphology, intensity, and texture. Nevertheless, the absence of statistically significant individual features after correction for multiple comparisons, together with the limited cohort size, warrants cautious interpretation of the multivariable results and confirmation in larger independent datasets.

However, the proposed model could support multidisciplinary decision-making and patient selection in clinical practice if externally validated. Before it can be implemented in routine clinical practice, its performance and generalizability must be confirmed through external validation in large, independent multicentre cohorts encompassing diverse patient populations, imaging equipment, and acquisition protocols. Such validation is essential to establish the robustness, reproducibility, and clinical utility of the model across different institutions.

## 5. Conclusions

In conclusion, this study shows that using a radiomics-based machine-learning approach with preoperative CT imaging can accurately predict the pathological response to neoadjuvant chemoimmunotherapy in patients with operable locally advanced NSCLC. These findings support the integration of radiomics into preoperative evaluation workflows, enabling more precise patient selection and the tailoring of surgical strategies, thus advancing personalized treatment approaches. However, due to the small sample size and retrospective nature of the analysis, these results are preliminary, and require validation in larger prospective cohorts before they can be recommended for routine clinical use.

## Figures and Tables

**Table 1:** Pre-operative baseline characteristics.

Variables	Overall (*n* = 29)	pCR (*n* = 9)	Non-pCR (*n* = 20)	*p* Value
Age (mean ± SD)	65.45 ± 11.2	69.78 ± 5.61	63.50 ± 12.57	0.1657
Sex (*n*, %)				1.000
F	13 (44.8%)	4 (44.4%)	9 (45%)	
M	16 (55.2%)	5 (55.6%)	11 (55%)	
Smoking status				0.633
Ex	23 (79.3%)	8 (88.9%)	15 (75%)	
Current	6 (20.7%)	1 (11.1%)	5 (25%)	
Histology				0.158
Adenocarcinoma	22 (75.9%)	5 (55.6%)	17 (85%)	
Squamous	7 (24.1%)	4 (44.4%)	3 (15%)	
Tumour location				0.749
RUL	10 (34.5%)	4 (44.4%)	6 (30%)	
ML	2 (6.9%)	1 (11.1%)	1 (5%)	
RLL	5 (17.2%)	2 (22.3%)	3 (15%)	
LUL	6 (20.7%)	1 (11.1%)	5 (25%)	
LLL	6 (20.7%)	1 (11.1%)	5 (25%)	
Clinical stage				1.000
IIIA	14 (48.3%)	4 (44.4%)	10 (50%)	
IIIB	15 (51.7%)	5 (55.6%)	10 (50%)	
Nodal status (pre)				0.276
N0	5 (17.2%)	3 (33.3%)	2 (10%)	
N1	6 (20.7%)	2 (22.3%)	4 (20%)	
N2	18 (62.1%)	4 (44.4%)	14 (70%)	
Tumour size	4.27 ± 1.6	4.61 ± 1.7	4.05 ± 1.5	0.373
PDL1				1.000
<1%	3 (10.4%)	1 (11.2%)	2 (10%)	
1–50%	13 (44.8%)	4 (44.4%)	9 (45%)	
>50%	13 (44.8%)	4 (44.4%)	9 (45%)	
Clinical response				1.000
Yes	23 (79.3%)	7 (77.7%)	16 (80%)	
no	6 (20.7%)	2 (22.3%)	4 (20%)	
Time NA(days) to surgery	35 ± 2.5	33 ± 1.7	34 ± 2.1	0.221

**Table 2:** Model of Multi-Layer Perceptron classification performance in term of ROC-AUC, Accuracy, Sensitivity, Specificity, PPV, NPV, and F1 score, corresponding to 95% confidence interval. Performance is reported for training, Validation, and Internal Testing.

	Training	Validation	Internal Testing
ROC-AUC (%) [95%]	99% [98–99]	93% [90–96]	92% [81–100]
Accuracy (%) [95%]	98% [97–99]	87% [84–90]	91% [84–98]
Sensitivity (%) [95%]	97% [95–99]	83% [77–89]	90% [79–100]
Specificity (%) [95%]	99% [98–100]	90% [86–93]	92% [84–100]
PPV (%) [95%]	97% [95–99]	86% [82–91]	87% [74–100]
NPV (%) [95%]	99% [98–100]	94% [92–96]	96% [91–100]
F1 Score (%) [95%]	97% [95–99]	76% [70–82]	87% [75–98]

**Table 3:** Model of Multi-Layer Perceptron classification performance in terms of ROC-AUC, Accuracy, Sensitivity, Specificity, PPV, NPV, and F1 score. Performance is reported for internal testing set, stratified by patient sex.

	Internal Testing (50% Threshold)—Female	Internal Testing (50% Threshold)—Male	Internal Testing (40% Threshold)—Female	Internal Testing (40% Threshold)—Male
ROC-AUC (%) [95%]	100	80	100	80
Accuracy (%) [95%]	93	93	87	86
Sensitivity (%) [95%]	100	75	100	75
Specificity (%) [95%]	90	100	80	90
PPV (%) [95%]	83	100	71	75
NPV (%) [95%]	100	91	100	90
F1 Score (%) [95%]	91	86	83	75

**Table 4:** Top 40 radiomic predictors ranked according to statistical significance. Median values, 95% confidence intervals, Wilcoxon rank-sum test results, and Bonferroni-adjusted *p*-values are reported for complete responders and non-complete responders.

	Feature Family	Feature Nomenclature	Median in the Negative Class [95% CI]	Median in the Positive Class [95% CI]	Uncorrected *p*-Value	Corrected *p*-Value
I	Deep Learning-Based	DeepFeature 443	2.10 × 10^−3^ [−7.61 × 10^−3^–1.18 × 10^−2^]	7.33 × 10^−2^ [3.38 × 10^−2^–1.13 × 10^−1^]	<0.05	0.47
II	Deep Learning-Based	DeepFeature 1376	0.00 × 10^0^ [−3.19 × 10^−3^–3.19 × 10^−3^]	7.72 × 10^−2^ [1.05 × 10^−3^–1.53 × 10^−1^]	<0.05	1.00
III	Deep Learning-Based	DeepFeature 1927	1.04 × 10^−3^ [−1.71 × 10^−2^–1.92 × 10^−2^]	4.00 × 10^−2^ [9.63 × 10^−4^–7.91 × 10^−2^]	<0.05	1.00
IV	Deep Learning-Based	DeepFeature 1365	0.00 × 10^0^ [−8.98 × 10^−4^–8.98 × 10^−4^]	0.00 × 10^0^ [0.00 × 10^0^–0.00 × 10^0^]	0.05	1.00
V	Deep Learning-Based	DeepFeature 634	0.00 × 10^0^ [0.00 × 10^0^–0.00 × 10^0^]	0.00 × 10^0^ [−3.36 × 10^−3^–3.36 × 10^−3^]	0.05	1.00
VI	Deep Learning-Based	DeepFeature 636	2.29 × 10^−1^ [1.05 × 10^−1^–3.52 × 10^−1^]	4.03 × 10^−1^ [1.84 × 10^−1^–6.21 × 10^−1^]	0.06	1.00
VII	Deep Learning-Based	DeepFeature 574	0.00 × 10^0^ [0.00 × 10^0^–0.00 × 10^0^]	0.00 × 10^0^ [−1.62 × 10^−2^–162 × 10^−2^]	0.06	1.00
VIII	Deep Learning-Based	DeepFeature 55	0.00 × 10^0^ [0.00 × 10^0^–0.00 × 10^0^]	1.63 × 10^−2^ [−5.42 × 10^−2^–8.96 × 10^−2^]	0.08	1.00
IX	Deep Learning-Based	DeepFeature 902	0.00 × 10^0^ [−1.71 × 10^−3^–1.71 × 10^−3^]	0.00 × 10^0^ [0.00 × 10^0^–0.00 × 10^0^]	0.11	1.00
X	Deep Learning-Based	DeepFeature 1196	0.00 × 10^0^ [−2.58 × 10^−3^–1.71 × 10^−3^]	4.26 × 10^−3^ [−3.08 × 10^−3^–1.16 × 10^−2^]	0.12	1.00
XI	Deep Learning-Based	DeepFeature 1586	0.00 × 10^0^ [−9.89 × 10^−4^–9.89 × 10^−4^]	0.00 × 10^0^ [0.00 × 10^0^–0.00 × 10^0^]	0.12	1.00
XII	Grey-level Run Length Matrix	Log High Grey-Level Run Emphasis	110 × 10^3^ [9.88 × 10^2^–1.21 × 10^3^]	9.88 × 10^2^ [9.13 × 10^2^–106 × 10^3^]	0.18	1.00
XIII	Deep Learning-Based	DeepFeature 683	2.80 × 10^−2^ [4.15 × 10^−3^–4.01 × 10^−3^]	0.00 × 10^0^ [−2.69 × 10^−2^–2.69 × 10^−2^]	0.19	1.00
XIV	Deep Learning-Based	DeepFeature 39	0.00 × 10^0^ [−4.01 × 10^−3^–4.01 × 10^−3^]	0.00 × 10^0^ [0.00 × 10^0^–0.00 × 10^0^]	0.20	1.00
XV	Deep Learning-Based	DeepFeature 1295	3.78 × 10^−2^ [9.14 × 10^−3^–6.64 × 10^−2^]	5.80 × 10^−3^ [2.11 × 10^−2^–3.27 × 10^−2^]	0.27	1.00
XVI	Deep Learning-Based	DeepFeature 275	0.00 × 10^0^ [0.00 × 10^0^–0.00 × 10^0^]	0.00 × 10^0^ [−1.06 × 10^−2^–1.06 × 10^−2^]	0.30	1.00
XVII	Deep Learning-Based	DeepFeature 1794	0.00 × 10^0^ [0.00 × 10^0^–0.00 × 10^0^]	0.00 × 10^0^ [−1.78 × 10^−3^–1.78 × 10^−3^]	0.30	1.00
XVIII	Intensity Histogram	Square Kurtosis	2.32 × 10^1^ [1.26 × 10^−2^–1.12 × 10^−2^]	2.87 × 10^1^ [9.30 × 10^−1^–5.64 × 10^1^]	0.31	1.00
XIX	Deep Learning-Based	DeepFeature 1289	0.00 × 10^0^ [−1.12 × 10^−2^–1.12 × 10^−2^]	1.17 × 10^−2^ [−4.40 × 10^−3^–2.78 × 10^−2^]	0.33	1.00
XX	Deep Learning-Based	DeepFeature 1474	0.00 × 10^0^ [−3.26 × 10^−2^–3.26 × 10^−2^]	7.54 × 10^−3^ [−3.53 × 10^−5^–1.51 × 10^−2^]	0.35	1.00
XXI	Deep Learning-Based	DeepFeature 1473	4.55 × 10^−3^ [−5.68e–03–1.48 × 10^−2^]	0.00 × 10^0^ [−3.44 × 10^−3^–3.44 × 10^−3^]	0.37	1.00
XXII	Deep Learning-Based	DeepFeature 1895	3.48 × 10^−3^ [−5.68 × 10^−3^–1.27 × 10^−2^]	0.00 × 10^0^ [−1.25 × 10^−2^–3.442-03]	0.37	1.00
XXIII	Grey Level Size Zone Matrix	Squareroot Grey-Level Variance	2.00 × 10^2^ [1.76 × 10^2^–2.23 × 10^2^]	1.89 + 02 [1.70 × 10^2^–2.09 × 10^2^]	0.38	1.00
XXIV	Deep Learning-Based	DeepFeature 1931	2.66 × 10^−2^ [1.27 × 10^−2^–4.05 × 10^−2^]	1.08 × 10^−3^ [−3.37 × 10^−2^–3.58 × 10^−2^]	0.40	1.00
XXV	Grey Level Co-occurrence Matrix	Second Measure of Information Correlation	7.74 × 10^−1^ [7.34-01–8.13 × 10^−1^]	7.71 × 10^−1^ [7.23 × 10^−1^–8.19 × 10^−1^]	0.46	1.00
XXVI	Deep Learning-Based	DeepFeature 995	0.00 × 10^0^ [−7.38 × 10^−4^–7.38 × 10^−4^]	0.00 × 10^0^ [−2.93 × 10^−3^–2.93]	0.49	1.00
XXVII	Deep Learning-Based	DeepFeature 1820	6.13 × 10^−2^ [1.84 × 10^−2^–1.04 × 10^−1^]	1.02 × 10^−1^ [1.07 × 10^−3^–2.04 × 10^−1^]	0.52	1.00
XXVIII	Intensity Based Statistics	Exponential Minimum	1.15 × 10^−1^ [8.71 × 10^−2^–1.44 × 10^−1^]	1.05 × 10^−1^ [7.04 × 10^−2^–1.40 × 10^−1^]	0.59	1.00
XXIX	Deep Learning-Based	DeepFeature 436	0.00 × 10^0^ [−4.64 × 10^−3^–4.64 × 10^−3^]	0.00 × 10^0^ [−7.07 × 10^−4^–7.07 × 10^−4^]	0.66	1.00
XXX	Deep Learning-Based	DeepFeature 332	0.00 × 10^0^ [0.00 × 10^0^–0.00 × 10^0^]	0.00 × 10^0^ [0.00 × 10^0^–0.00 × 10^0^]	0.72	1.00
XXXI	Neighbouring Grey Level Dependence Matrix	Exponential Dependence Count Energy	7.28 × 10^−3^ [6.61 × 10^−3^–7.94 × 10^−3^]	7.70 × 10^−3^ [6.12 × 10^−3^–9.28 × 10^−3^]	0.72	1.00
XXXII	Grey Level Size Zone matrix	Gradient Zone Size Non-Uniformity	2.73 × 10^3^ [4.31 × 10^2^–5.04 × 10^3^]	2.59 × 10^3^ [1.87 × 10^3^–4.10 × 10^3^]	0.76	1.00
XXXIII	Grey Level co-occurrence matrix	Cluster Prominence	2.26 × 10^3^ [1.17 × 10^3^–3.35 × 10^3^]	2.59 × 10^3^ [1.07 × 10^3^–4.10 × 10^3^]	0.80	1.00
XXXIV	Deep Learning-Based	DeepFeature 511	0.00 × 10^0^ [−1.38 × 10^−3^–1.38 × 10^−3^]	0.00 × 10^0^ [−1.53 × 10^−2^–1.53 × 10^−2^]	0.83	1.00
XXXV	Grey Level Run Length Matrix	High Grey-Level Run Emphasis	1.70 × 10^3^ [1.61 × 10^3^–1.79 × 10^3^]	1.67 × 10^3^ [1.58 × 10^3^–1.77 × 10^3^]	0.87	1.00
XXXVI	Deep Learning-Based	DeepFeature 1781	5.12 × 10^−3^ [−1.54 × 10^−3^–1.18 × 10^−2^]	3.83 × 10^−3^ [−3.12 × 10^−3^–1.08 × 10^−2^]	0.92	1.00
XXXVII	Deep Learning-Based	DeepFeature 1321	5.13 × 10^−3^ [−1.54 × 10^−3^–1.18 × 10^−2^]	3.83 × 10^−3^ [3.12 × 10^−3^–1.08 × 10^−2^]	0.94	1.00
XXXVIII	Morphology	Compactness1	2.27 × 10^−2^ [1.76 × 10^−2^–2.78 × 10^−2^]	1.86 × 10^−2^ [1.16 × 10^−2^–2.55 × 10^−2^]	0.94	1.00
XXXIX	Grey Level Size Zone Matrix	Gradient Small Zone Low Grey-Level Emphasis	1.40 × 10^−2^ [1.06 × 10^−2^–1.73 × 10^−2^]	1.61 × 10^−2^ [1.13 × 10^−2^–2.09 × 10^−2^]	0.94	1.00
XL	Deep Learning-Based	DeepFeature 1343	0.00 × 10^0^ [−1.50 × 10^−3^–1.50 × 10^−3^]	0.00 × 10^0^ [−4.23 × 10^−3^–4.23 × 10^−3^]	1.00	1.00

## Data Availability

All the available data are included in the manuscript.

## Abbreviations

The following abbreviations are used in this manuscript:

NA	Neo-Adjuvant
ICI-CHT	Immune-Checkpoint-Inhibitors Chemotherapy
CT	Computed Tomography
NSCLC	Non-Small-Cell Lung Cancer
ITT	Intention-To-Treat
PDL-1	Programmed Death-Ligand 1
AI	Artificial Intelligence
MRI	Magnetic Resonance Imaging
PET	Positron Emission Tomography
RECIST	Response Evaluation Criteria in Solid Tumours
PCR	Pathological Complete Response
VOI	Volume Of Interest
EBUS	Endobronchial Ultrasound
IBSI	Biomarker Standardization Initiative
ADASYN	Adaptive Synthetic Sampling
PCA	Principal Component Analysis
FDR	Fisher Discriminant Ratio
ROC-AUC	Receiver operating characteristic area under the curve
PPV	Positive Predictive Value
NPV	Negative Predictive Value
ECE	Expected Calibration Error
BS	Brier Score
TBNA	Trans-Bronchial Needle Aspiration
MPR	Major Pathologic Response
SD	Standard Deviation

## References

[1] Bray F., Ferlay J., Soerjomataram I., Siegel R.L., Torre L.A., Jemal A. (2018). Global cancer statistics 2018: GLOBOCAN estimates of incidence and mortality worldwide for 36 cancers in 185 countries. CA Cancer J. Clin..

[2] Provencio M., Nadal E., González-Larriba J.L., Martínez-Martí A., Bernabé R., Bosch-Barrera J., Casal-Rubio J., Calvo V., Insa A., Ponce S. (2023). Perioperative Nivolumab and Chemotherapy in Stage III Non–Small-Cell Lung Cancer. N. Engl. J. Med..

[3] Desai A.P., Adashek J.J., Reuss J.E., West H., Mansfield A.S. (2023). Perioperative immune checkpoint inhibition in early-stage non-small cell lung cancer: A review. JAMA Oncol..

[4] Forde P.M., Spicer J., Lu S., Provencio M., Mitsudomi T., Awad M.M., Felip E., Broderick S.R., Brahmer J.R., Swanson S.J. (2022). Neoadjuvant Nivolumab plus Chemotherapy in Resectable Lung Cancer. N. Engl. J. Med..

[5] Lin Q., Wu H.J., Song Q.S., Tang Y.K. (2022). CT-based radiomics in predicting pathological response in non-small cell lung cancer patients receiving neoadjuvant immunotherapy. Front. Oncol..

[6] Scrivener M., de Jong E.E.C., van Timmeren J.E., Pieters T., Ghaye B., Geets X. (2016). Radiomics applied to lung cancer: A review. Transl. Cancer Res..

[7] Pan F., Feng L., Liu B., Hu Y., Wang Q. (2023). Application of radiomics in diagnosis and treatment of lung cancer. Front. Pharmacol..

[8] Kim H.K., Heo M.H., Lee H.S., Sun J.-M., Lee S.-H., Ahn J.S., Park K., Ahn M.-J. (2017). Comparison of RECIST to immune-related response criteria in patients with non-small cell lung cancer treated with immune-checkpoint inhibitors. Cancer Chemother. Pharmacol..

[9] Barabino E., Rossi G., Pamparino S., Fiannacca M., Caprioli S., Fedeli A., Zullo L., Vagge S., Cittadini G., Genova C. (2022). Exploring Response to Immunotherapy in Non-Small Cell Lung Cancer Using Delta-Radiomics. Cancers.

[10] Siciliani A., Guido G., De Santis D., Bracci B., Masci B., Faggiano A., Mikovic N., Paravani P., Martiradonna M., Palmeri F. (2025). Role of Chest CT Radiomics in Differentiating Tumorlets and Granulomas: A Preliminary Study. J. Clin. Med..

[11] Zwanenburg A., Vallières M., Abdalah M.A., Aerts H.J.W.L., Andrearczyk V., Apte A., Ashrafinia S., Bakas S., Beukinga R.J., Boellaard R. (2020). The Image Biomarker Standardization Initiative: Standardized Quantitative Radiomics for High-Throughput Image-based Phenotyping. Radiology.

[12] Dacic S., Travis W., Redman M., Saqi A., Cooper W.A., Borczuk A., Chung J.-H., Glass C., Lopez J.M., Roden A.C. (2023). International Association for the Study of Lung Cancer Study of Reproducibility in Assessment of Pathologic Response in Resected Lung Cancers After Neoadjuvant Therapy. J. Thorac. Oncol..

[13] Goldstraw P., Chansky K., Crowley J., Rami-Porta R., Asamura H., Eberhardt W.E., Nicholson A.G., Groome P., Mitchell A., Bolejack V. (2016). The IASLC Lung Cancer Staging Project: Proposals for Revision of the TNM Stage Groupings in the Forthcoming (Eighth) Edition of the TNM Classification for Lung Cancer. J. Thorac. Oncol..

[14] A Waser N., Quintana M., Schweikert B., E Chaft J., Berry L., Adam A., Vo L., Penrod J.R., Fiore J., A Berry D. (2024). Pathological response in resectable non–small cell lung cancer: A systematic literature review and meta-analysis. JNCI Cancer Spectr..

[15] Linden P.A., Martin L.W., Stiles B., Opitz I., Chen H., Spicer J., Lanuti M., Tong B.C., Liberman M., Bestvina C.M. (2026). The 2026 American Association for Thoracic Surgery Expert Consensus Document: Evaluation and management of N2+ non–small cell lung cancer. J. Thorac. Cardiovasc. Surg..

[16] Liberman M., Jones D.R., Wakelee H., Gao S., Halmos B., Nadal E., Łowczak A., Reck M., Novello S., Matias D. (2026). Clinical Characteristics and Surgical Outcomes of Patients Receiving Perioperative Pembrolizumab in KEYNOTE-671. Ann. Thorac. Surg..

[17] Heymach J., Reck M., Mitsudomi T., Taube J.M., Spira A.I., Chaft J.E., Doherty G.J., Mann H., Fouad T.M., Harpole D.H. (2024). Outcomes with perioperative durvalumab (D) in pts with resectable NSCLC and baseline N2 lymph node involvement (N2 R-NSCLC): An exploratory subgroup analysis of AEGEAN. J. Clin. Oncol..

[18] Verma S., Breadner D., Mittal A., Palma D.A., Nayak R., Raphael J., Vincent M. (2024). An Updated Review of Management of Resectable Stage III NSCLC in the Era of Neoadjuvant Immunotherapy. Cancers.

[19] Zhou L., Zhang M., Li R., Xue J., Lu Y. (2020). Pseudoprogression and hyperprogression in lung cancer: A comprehensive review of literature. J. Cancer Res. Clin. Oncol..

[20] Lambin P., Rios-Velazquez E., Leijenaar R., Carvalho S., van Stiphout R.G.P.M., Granton P., Zegers C.M.L., Gillies R., Boellard R., Dekker A. (2012). Radiomics: Extracting more information from medical images using advanced feature analysis. Eur. J. Cancer.

[21] Wang F., Yang H., Chen W., Ruan L., Jiang T., Cheng L., Jiang H., Fang M. (2024). A combined model using pre-treatment CT radiomics and clinicopathological features of non-small cell lung cancer to predict major pathological responses after neoadjuvant chemoimmunotherapy. Curr. Probl. Cancer.

[22] Bortolotto C., Lancia A., Stelitano C., Montesano M., Merizzoli E., Agustoni F., Stella G., Preda L., Filippi A.R. (2021). Radiomics features as predictive and prognostic biomarkers in NSCLC. Expert Rev. Anticancer Ther..

